# Early postoperative administration of probiotics versus placebo in elderly patients undergoing elective colorectal surgery: a double-blind randomized controlled trial

**DOI:** 10.1186/1471-2482-13-S2-S57

**Published:** 2013-10-08

**Authors:** Gianluca Pellino, Guido Sciaudone, Giuseppe Candilio, Antonio Camerlingo, Rosa Marcellinaro, Serena De Fatico, Federica Rocco, Silvestro Canonico, Gabriele Riegler, Francesco Selvaggi

**Affiliations:** 1 Unit of General and Geriatric Surgery, Second University of Naples, Italy; 2 Unit of Gastroenterology, Second University of Naples, Italy

## Abstract

**Background:**

Perioperative prophylactic antibiotic treatment significantly influences intestinal microflora, resulting in impaired bowel functioning in some patients, sometimes requiring further investigations. This may lead to a worse health-related quality of life (HRQoL). Probiotics administrated in the early post-operative period may help avoiding such nuisances in older people.

**Methods:**

We prospectively enrolled patients undergoing laparoscopic colorectal surgery aged over 70 years between 2005 and 2012. The study was approved by IRB. All patients received perioperative antibiotic treatment. Patients were randomized to one of two treatment arms: Group A patients received probiotics after surgery (VSL#3, VSL Pharmaceuticals, Inc. USA - 900 × 10^9 ^daily, while Group B patients received a Placebo (cornstarch). Patients were further divided in sub-groups whether ileo-caecal valve was spared or not. Patients were followed-up every 7 days for 4 weeks. Patients daily annotated bowel frequency, stool shape and consistency - according to Bristol's scale. HRQoL was assessed every week by means of SF-36 questionnaire.

**Results:**

Group A included 10 while Group B included 8 patients. One patient in each group experienced a postoperative complication. Group A patients had fewer bowel movements than controls, during every week. Stool consistency was higher in patients undergoing resections including ileo-caecal valve receiving VSL#3. HRQoL gradually increased in both groups; Group A patients had higher "social functioning" item scores at week 1 and 4 than controls.

**Conclusions:**

Elderly patients undergoing resection of ileo-caecal valve may benefit from an early probiotics administration pathway after perioperative antibiotic treatment.

## Background

Intestinal microflora is profoundly influenced by antibiotic therapy needed peri-operatively in patients undergoing large bowel resections. The effects of short antibiotic therapy on gut microbiota can last more than 24 months [1]. Elderly patients operated on may often suffer from functional intestinal nuisances in the postoperative period, which may require further investigations and treatments. The possibility of modulating intestinal microflora by means of probiotic formulations could potentially lead to a better functioning of gut mucosal barrier, resulting in reduced septic complications. Also, bowel control and health-related quality of life (HRQoL) may be improved.

Aim of our study was to investigate whether the administration of probiotics in the early postoperative period after large bowel resection can facilitate the restoration of bowel function and reduce symptoms in elderly patients.

## Methods

This is a prospective double blind randomized trial on patients aged over 70 years undergoing laparoscopic colonic resection in our Unit between 2005 and January 2012. The study was approved by IRB and all patients were included after signing an informed consent. Patients were randomly assigned to Group A (antibiotic + probiotic) or Group B (antibiotic + placebo). In each group, patients were further divided in sub-groups 1 and 2 whether surgery preserved or not ileocaecal valve, respectively. After the discharge, patients were seen in outpatient settings by a resident every 7 days for 4 weeks. Neither patients nor residents knew which treatment (probiotic/placebo) was being administered.

### Exclusion criteria

Dukes D colorectal cancer

Inflammatory bowel diseases

Need for temporary or definitive oostomy

Allergy to antibiotics/probiotics/placebo used in the study

### Perioperative antibiotic therapy

Patients received i.v. cefotaxime 1 g and i.v. metronidazole 1 g within 30 minutes from surgery, and continued on i.v. cefotaxime 1g t.i.d. and i.v. metronidazole 500 mg t.i.d. for no more than 24 hours postoperatively.

### Probiotic/placebo therapy

Group A patients received probiotics (VSL#3, VSL Pharmaceuticals, Inc. USA) at the dose of 900 × 10^9 ^daily, divided in two administrations from one day after discontinuation of antibiotic through four weeks. The probiotic composition is the following: *Streptococcus thermophilus *DSM 24731, *Bifidobacteria *(*B. longum *DSM 24736, *B. breve *DSM 24732, *B. infantis *DSM 24737), *Lattobacilli *(*L. acidophilus *DSM 24735, *L. plantarum *DSM 24730, *L. paracasei *DSM 24733, *L. delbrueckii *subsp. *bulgaricus *DSM 24734); maltose; silicium dioxide. Group B patients received the Placebo composed of cornstarch (base ingredient for probiotic product), indistinguishable from VSL#3, in a similar fashion.

### Bowel function assessment

During probiotic/placebo treatment patients were requested to complete a daily report annotating: bowel movement/s, body temperature, stool shape and consistency. Stool characters were defined according to Bristol scale [2], a 7-point scale in which stools were scored according to cohesion and surface cracking, as follows: 1, separate hard lumps (like nuts); 2, sausage-shaped, but lumpy; 3, like a sausage or snake, but with cracks on its surface; 4, like a sausage or snake, and smooth and soft; 5, soft blobs with a clear cut edge; 6, fluffy pieces with ragged edges and mushy; and 7, watery with no solid pieces).

### Quality of life assessment

Patients completed the SF-36 questionnaire every week during probiotic/placebo treatment.

### Statistical analysis

We enrolled all consecutive patients undergoing surgery in the study period fitting inclusion criteria. Data are presented as mean ± SD. Categorical variable were compared by means of Fisher's exact test; for continuous parameters Student's *t *test was used.

## Results

**Figure 1:**
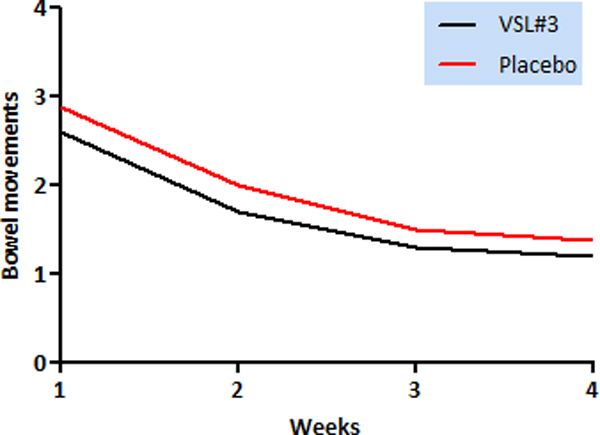
**Frequency of bowel movements in Group A (VSL#3) vs Group B (Placebo)**. Patients treated with probiotics had less mean bowel moments/day at each week but the observation did not reach statistical significance (*p *= 0.06)

Group A included 10 patients (5 male); Group B included 8 patients (4 male). Patients characteristics and surgical parameters are reported in Table 1. One patient in each group experienced a postoperative complication, managed conservatively. Irrespective of ileo-caecal valve preservation, Group A patients had fewer bowel movements than controls, during every week (Figure 1).

**Table 1:** Characteristics of patients and surgical details

Characteristics	Group A VSL#3 (n 10)	Group B placebo (n 8)	*P* *value*
Age, yr (± SD)	71.5 (±2.1)	72.9 (±1.6)	NS

BMI, kg/m^2 ^(± SD)	21.5 (±3.9)	20.4 (±2.7)	NS

Gender, n (M/F)	5/5	4/4	NS

Comorbidities, n (%)	8 (80)	7 (87.5)	NS

ASA score ≥ III, n (%)	1 (10)	1 (12.5)	NS

Operative time. min (± SD)	160.3 (±33.2)	155.8 (±41.1)	NS

Procedure performed Right hemicolectomy, n (%) Left hemicolectomy, n (%)	4 (40) 6 (60)	4 (50) 4 (50)	NS

Complications*, n (%)	1 (10)	1 (12.5)	NS

Length of stay, day (± SD)	12.0 (± 8.3)	13.5 (± 4.8)	NS

SD: standard deviation

BMI: body mass index

ASA: American Society of Anaesthesiologists

* Group A: Prolonged ileus managed conservatively

Group B: Haemorrhage managed conservatively with blood transfusion

**Figure 2:**
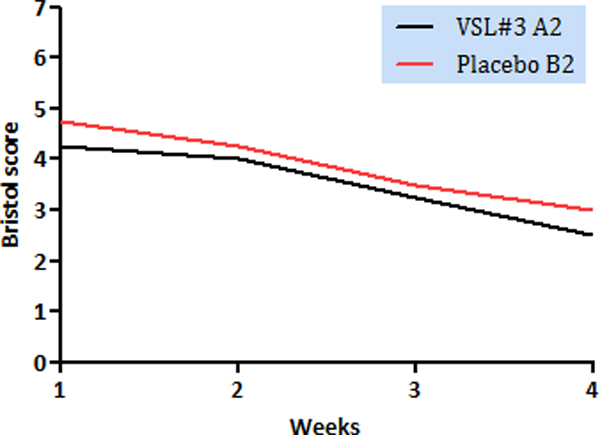
**Bristol scores in subgroups of patients undergoing ileocaecal resections**. Group A2 (VSL#3) patients had significantly higher consistency stools than Group B2 (Placebo) patients throughout 4 weeks (*p *= 0.03)

No differences were observed concerning overall stool consistency; however it was higher in patients undergoing ileocolic resections including ileo-caecal valve receiving VSL#3 (Figure 2).

No febrile episodes (body temperature ≥ 37.3° C) were recorded.

**Figure 3:**
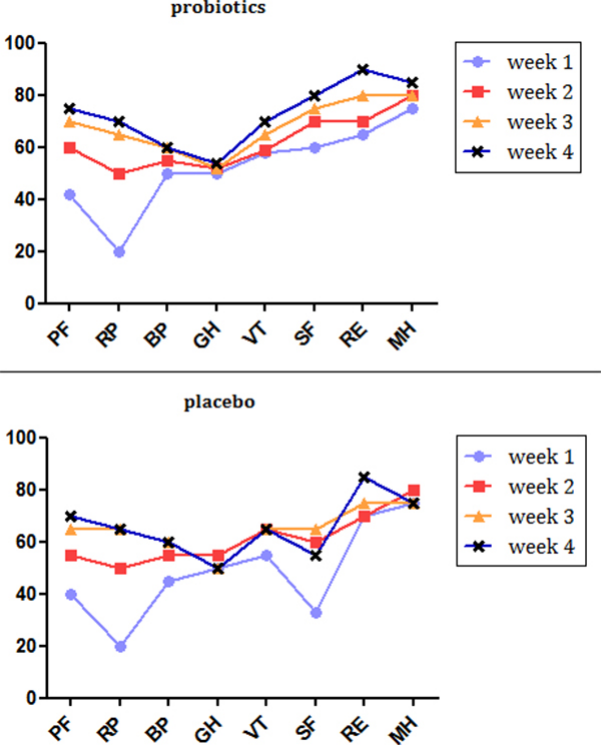
**SF-36 quality of life items scores in Group A (VSL#3) vs Group B (Placebo)**. No differences were observed overall; at 1- and 4-week follow-up, Group A patients showed significantly better social functioning (SF) than controls (*p *= 0.04 and *p *= 0.002).

Both groups showed a gradual increase in HRQoL from first through fourth week; with respect to item "social functioning", group A patients showed significantly higher score at week 1 and 4 than patients receiving placebo (Figure 3).

No adverse effects to VSL#3 or placebo were observed.

## Discussion

Perioperative antibiotic therapy, although demandable, are responsible for relevant imbalances of intestinal microbiota [1,3]. This may lead to higher risk of infectious complications due to bacterial translocation. Also, the imbalance in components of gut microflora is responsible for intensified symptoms perception [1].

It has been reported that probiotic formulations are not effective to reduce the rate of postoperative septic complications in patients undergoing elective abdominal surgery [3]. However, literature lacks studies investigating the effect of probiotics administration in the early post-operative period on symptoms perception in elderly patients undergoing large bowel resection.

In our series, we observed a lower frequency of evacuations in patients receiving probiotics, although not statistically significant; stool consistency was slightly higher in patients receiving resection not preserving ileo-caecal valve treated with probiotics. HRQoL was comparable in both groups, but patients in the probiotic group achieved a better social functioning than controls at 1- and 4-week follow-up.

## Conclusions

Even if therapy with probiotics did not show a protective effect on postoperative complications, frail patients undergoing resection of ileo-caecal valve may benefit from an early probiotics administration pathway after perioperative antibiotic treatment. Bowel frequency may be lower, irrespective of the type of surgery. These observations support the usefulness of early probiotic administration in achieving a prompt restoration of function and return to everyday activities.

## List of Abbreviations used

HRQoL: health-related quality of life; IRB: internal review board; SF-36: short-form-36 questionnaire; SD: standard deviation.

## Competing interests

The authors declare that they have no competing interests.

## Authors' contributions

G.P.: conception and design, interpretation of data, given final approval of the version to be published

G.S.: acquisition of data, drafting the manuscript, given final approval of the version to be published

G.C.: acquisition of data, drafting the manuscript, given final approval of the version to be published

A.C.: acquisition of data, drafting the manuscript, given final approval of the version to be published

R.M.: acquisition of data, drafting the manuscript, given final approval of the version to be published

S.D.F.: acquisition of data, drafting the manuscript, given final approval of the version to be published

F.R.: acquisition of data, drafting the manuscript, given final approval of the version to be published

S.C.: critical revision, interpretation of data, given final approval of the version to be published

G.R.: conception and design, critical revision, given final approval of the version to be published

F.S.: conception and design, critical revision, given final approval of the version to be published

## Authors' information

GP: Resident in General Surgery at Second University of Naples.

GS: PhD, Researcher, Aggregate Professor of General Surgery at Second University of Naples.

GC: Resident in General Surgery at Second University of Naples.

AC: Medical Student (Research Assistant) at Second University of Naples.

RM: Medical Student (Research Assistant) at Second University of Naples.

SDF: M.D. (Research Assistant) at the Second University of Naples.

FR: Medical Student (Research Assistant) at Second University of Naples.

CS: Full Professor of General Surgery at Second University of Naples, Chief Division of General and Geriatric Surgery Second University of Naples.

GR: Associate Professor of Gastroenterology, Second University of Naples.

FS: Associate Professor of General Surgery at Second University of Naples.

## Declarations

Funding for this article has come from personal funds.

This article has been published as part of *BMC Surgery *Volume 13 Supplement 2, 2013: Proceedings from the 26th National Congress of the Italian Society of Geriatric Surgery. The full contents of the supplement are available online at http://www.biomedcentral.com/bmcsurg/supplements/13/S2
